# Trigeminal Trophic Syndrome Secondary to Refractory Trigeminal Neuralgia Treated with CyberKnife® Radiosurgery

**DOI:** 10.7759/cureus.7670

**Published:** 2020-04-14

**Authors:** Kita Sallabanda, Morena Sallabanda, Hernan Dario Barrientos, Iciar Santaolalla, Rafel Garcia

**Affiliations:** 1 Radiosurgery/Neurosurgery, Hospital Clinico Universitario San Carlos, Madrid, ESP; 2 Radiation Oncology, Cyberknife Centre, GenesisCare, Madrid, ESP; 3 Neurosurgery/Radiosurgery, Instituto Neurologico de Colombia, Medellin, COL; 4 Radiation Oncology/Medical Physics, GenesisCare, Madrid, ESP; 5 Radiation Oncology, Cyberknife Centre, Ruber Internacional, Madrid, ESP

**Keywords:** radiosurgery, functional, pain, trigeminal neuralgia, trigeminal trophic syndrome

## Abstract

Trigeminal trophic syndrome (TTS) is a rare condition in which there is the involvement of the skin innervated by branches of the trigeminal nerve. Because of an alteration in the sensory function of the trigeminal nerve, an exaggerated manipulation of the skin by the patient occurs, with secondary ulcers in the affected areas. They are usually unilateral and located mainly at the beginning of the nose wing. There are very few publications in the current literature, so it is in the interest of doctors to know this rare pathology.

## Introduction

Trigeminal trophic syndrome (TTS) is a rare pathology and classically presents with a triad of symptoms: trigeminal anesthesia, facial paresthesias, and crescent-shaped ulceration of the lateral nasal ala [1-2]. Sometimes, this syndrome occurs after significant injuries to the trigeminal nerve due to procedures and/or diseases that alter the structure of the nerve itself. As a consequence of an alteration in the sensory function of the trigeminal nerve, an exaggerated manipulation of the skin by the patient occurs, with secondary ulcers in the affected areas They are usually unilateral and located mainly at the beginning of the nose wing [1-4]. The expression of the pathology on the face is confusing and requires an exhaustive study with a differential diagnosis based on the biopsy.

We report a case of a patient who developed TTS as a consequence of refractory essential trigeminal neuralgia and underwent radiosurgery with the Cyberknife® (Accuray Incorporated, Sunnyvale, California) technique.

## Case presentation

**Figure 1:**
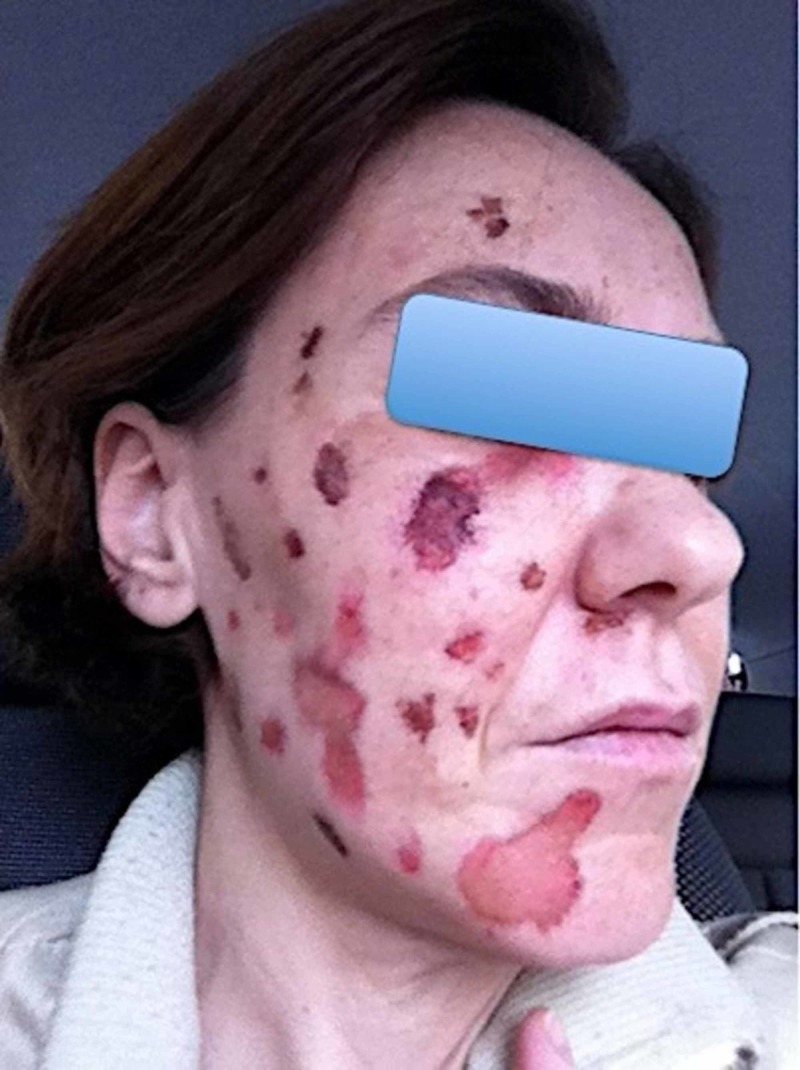
Patient image before SRS SRS: stereotactic radiosurgery

In March 2012, a 37-year-old, previously healthy woman started with essential right trigeminal neuralgia. Though pharmacological treatment was started, the patient did not improve. After one year of treatment, she presented with skin lesions on the face, along the areas innervated by the ipsilateral V cranial nerve. The patient was exhaustively studied, including an assessment by multiple specialists. Herpes and other infectious and dermatological diseases were ruled out by performing a biopsy of the skin and a pathology assessment. The lesions were increasing in size and were located in areas innervated by the three branches of right cranial nerve V. The lesions were painless and related to the longest period of painful symptoms (Figure 1). In July 2013, she underwent a Fogarty rhizotomy with no improvement. Subsequently, in January 2014, she underwent right suboccipital decompression with the Janetta microsurgical technique due to the clinical suspicion of vascular compression, without improvement. She continued to present very severe paroxysmal pain crises in the three branches of the right trigeminal daily, which did not respond to any type of treatment and continued with skin lesions. Although the patient did not report repeated manipulations in the innervation zone of the trigeminal nerve, a bandage was used on the face, seeking an improvement in the skin lesions, but the improvement was not as expected. The patient lost weight and when we first evaluated her, she was in a very altered psychological situation, due to both the pain and the expression of the disease on her face. Assessing the case and analyzing all the previous treatments in the context of neuralgia, it was decided to conduct radiosurgery using the CyberKnife technique in order to control pain.

Radiosurgical treatment

**Figure 2:**
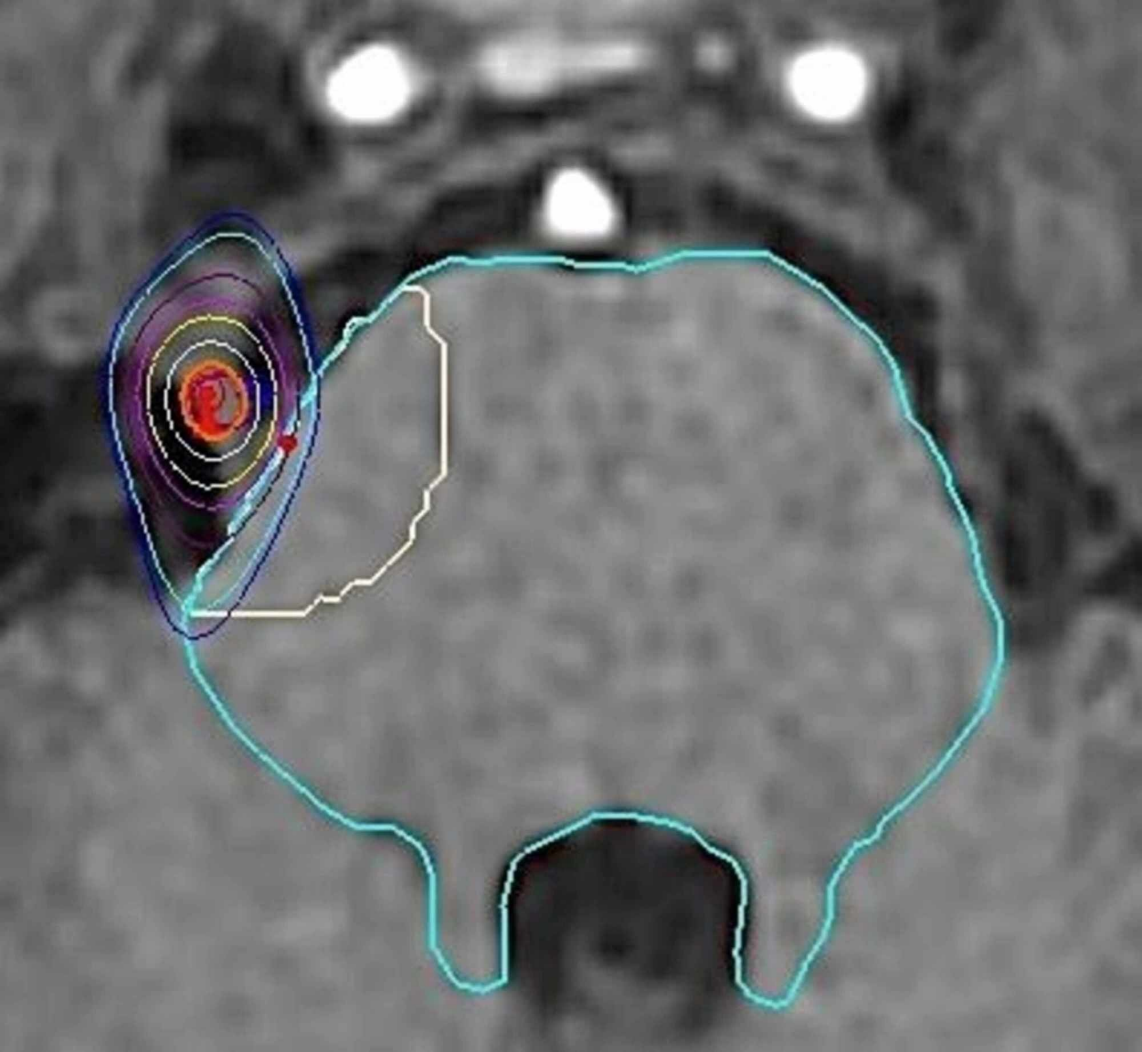
CyberKnife SRS treatment (MRI image) SRS: stereotactic radiosurgery; MRI: magnetic resonance imaging CyberKnife (Accuray Incorporated, Sunnyvale, California)

**Figure 3:**
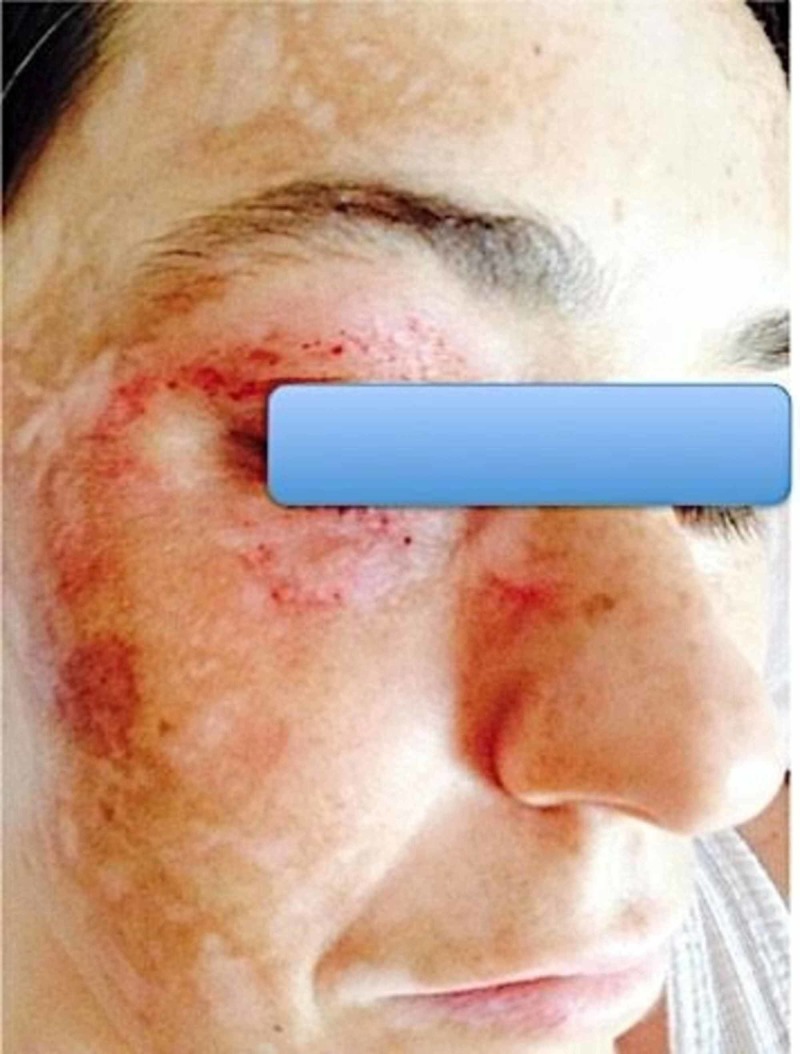
Patient image six months after SRS SRS: stereotactic radiosurgery

**Figure 4:**
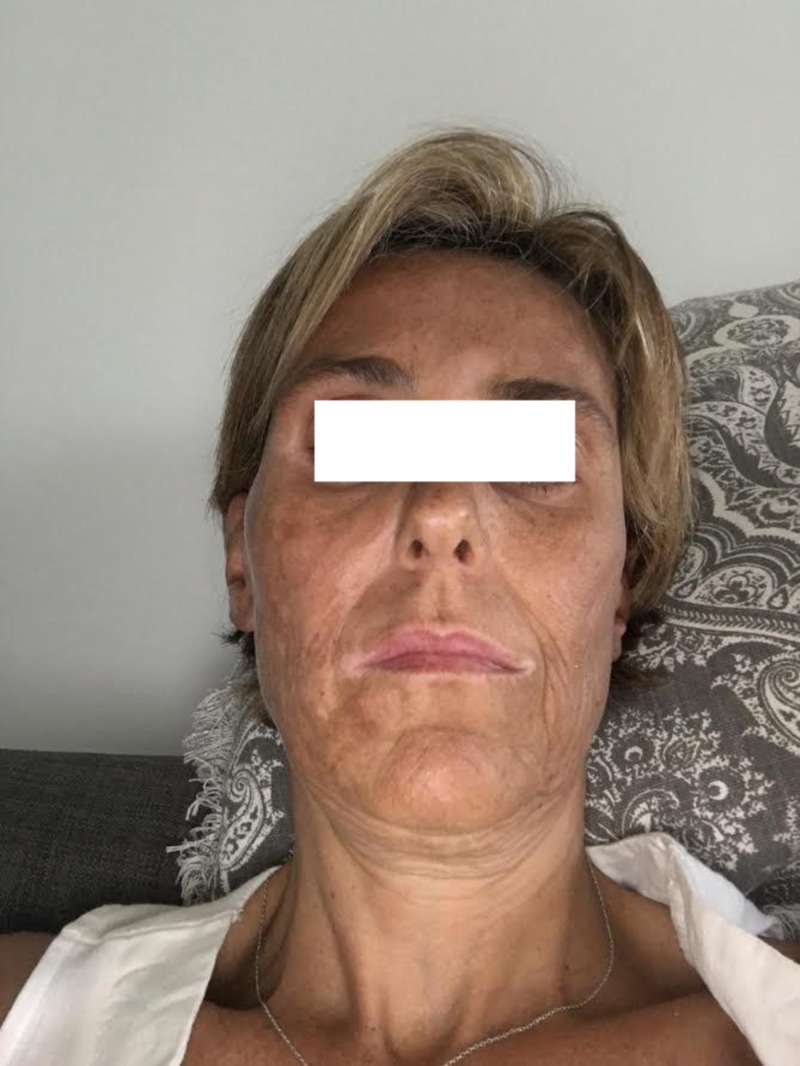
Patient image one year after SRS SRS: stereotactic radiosurgery

The patient is informed about the radiosurgical procedure and signs the consent. A reinforced immobilization thermoplastic mask is made, which has a greater thickness than the typical mask, to increase its rigidity and further decrease the possibility of head movement in order to achieve greater precision. Planning neuroimaging is performed: a simple CT with 1.2 mm fine cuts with the immobilization mask on and a simple MRI sequence T1, T2, and fast imaging employing steady-state acquisition (FIESTA) with fine cuts (Figure 2). Subsequently, images are merged in Multiplan software (Microsoft Corporation, Redmond, Washington). A volume of the trigeminal nerve was outlined, starting 3 mm apart from the brainstem and covering 5 mm of the trigeminal nerve, exceeding 1 mm on each side of the nerve. It was prescribed a dose of 60 Gy to an isodose of 80% of the outlined volume, provided that at least 50% of the volume received 70 Gy and seeking a maximum dose of 85 Gy. Isocentric reverse planning was performed with a brainstem constraint of V10 <0.5 cc and Dmax of 12 Gy (Figure 3). After radiosurgery treatment, the patient's neuropathic pain improved, decreasing on the analogous pain scale from 10 to 5 in the second week of treatment. Additionally, her skin lesions improved (Figures 3-4).

## Discussion

TTS, also reported as neurotropic trigeminal ulceration, trigeminal neuropathy with ulceration of the nasal wing, or trophic ulceration of the nasal wing, was initially described in 1933 by Loveman and McKenzie [5-9]. It is characterized by trigeminal anesthesia, facial paresthesia, and painless ulceration in the region of the face innervated by the trigeminal nerve, mainly in the region of the wing of the nose, but it rarely can also appear in other sites innervated by trigeminal branches such as the scalp, forehead, and the skin area of the jaw. It is a rare entity, with only 182 cases reported worldwide from 1933 to 2012 [1,3]. There are very few publications in the current literature, so it is in the interest of physicians to know this rare pathology. It occurs as a consequence of cranial nerve dysfunction due to multiple causes. Treatment is complex and is supported in education self-care and pharmacological and, occasionally, surgical treatment. It can develop due to any type of involvement of the trigeminal nerve or other causes such as central and/or peripheral alterations or functional, ischemic, traumatic, or iatrogenic etiology. Brain stem infarcts, vestibular nerve schwannomas, meningiomas and gliomas, syringobulbia, trigeminal neuralgia and its therapeutic procedures, infectious causes due to mycobacteria and herpes viruses, as well as trauma, and radiotherapy procedures have been reported as etiology [1,3,10].

According to the classical theory, because of nerve involvement, there is a sensory alteration of the innervated face area with the consequent abnormal manipulation of the patient that leads to painless, characteristically progressive ulceration. In our case, manipulation of the skin might not completely support the ulcers since while the face was bandaged, improvement in the lesions could not be verified. The list of differential diagnoses is long, and all possible causes of facial ulcers should be ruled out, from basal cell and squamous cell carcinomas, herpetic ulceration, syphilis, Wegener's granulomatosis, and other rarer lesions. Treatment consists of avoiding manipulation of the patient, neuromodulators, and plastic surgery [4,8-9].

This is the first reported case of TTS secondary to trigeminal neuralgia that has been treated with radiosurgery. We treated a case of trigeminal neuralgia, which was the underlying disease with an improvement in secondary neuropathic and dermatological symptoms. See Figure 3.

Radiosurgical rhizotomy with CyberKnife has been reported as a safe and effective procedure in the treatment of trigeminal neuralgia refractory to conventional treatments and produces necrosis, mainly in the sensory fibers of the trigeminal nerve, cutting the pain circuit [10-12]. Ulcerations in the nasal region have been studied by different ear-nose-throat (ENT) and dermatology specialists without having been able to clarify the pathophysiology or the best way of treatment [13-14].

The question we face is whether the improvement of his trigeminal pain has also had an influence on the improvement of TTS.

The review of the literature and our own studies do not allow us to establish a direct relationship to date, and we consider that a more in-depth investigation is necessary. However, the improvement in the sensory alteration that trigeminal neuralgia leads to may have had an influence on the improvement of TTS [1-9,13-15].

## Conclusions

In this report, we present a case of TTS associated with facial lesions in a patient with trigeminal neuralgia. The patient received SRS to treat trigeminal neuralgia, with a good response in both pain and skin lesions at the same time. This is the first case published of satisfactory TTS treatment with CyberKnife radiosurgical rhizotomy of the trigeminal cranial nerve. The evidence of clinical improvement in TTS (ulcers on the face) after trigeminal-related pain improvement from SRS pushes us to further investigate the relationship between these two clinical entities by analyzing other similar cases.
